# Supplementary material for: Application of Magnesium and Calcium Sulfate on Growth and Physiology of Forage Crops under Long-Term Salinity Stress
Source: Plants (Basel). 2022 Dec 18;11(24):3576. doi: 10.3390/plants11243576 (PMC9785884; doi:10.3390/plants11243576)
Supplement: Supplementary file 1 [file plants-11-03576-s001.zip › plants-2099882-si.pdf]

Table S1. Ion concentrations (g/kg) in stem, root, and leaf of *T. pratense*, *F. arundinacea* under six treatments at 60 DAT and 120 DAT.

Species	Date	Treatments	Na			Ca			K			Mg			P		
			Stem	Root	Leaf	Stem	Root	Leaf	Stem	Root	Leaf	Stem	Root	Leaf	Stem	Root	Leaf
T. pratense	60 DAT	CON	2.07^{d}	1.93^{d}	0.86^{d}	17.55^{bc}	5.39^{c}	10.37^{c}	22.41^{bc}	11.38^{bc}	19.26^{c}	4.36^{ab}	3.81^{a}	3.12^{c}	7.28^{d}	11.45^{d}	24.54^{a}
		NaCl	4.38^{a}	3.61^{c}	1.74^{c}	19.40^{a}	4.30^{d}	17.34^{a}	23.35^{b}	12.83^{a}	21.31^{b}	4.62^{ab}	1.95^{b}	5.59^{a}	7.69^{d}	15.71^{a}	16.76^{b}
		1 MS	2.58^{c}	5.09^{b}	2.64^{b}	18.67^{ab}	6.55^{b}	14.73^{b}	22.44^{bc}	11.58^{ab}	22.96^{a}	4.43^{ab}	3.87^{a}	4.15^{b}	7.65^{d}	13.75^{b}	14.88^{d}
		2 MS	2.13^{d}	4.88^{b}	2.09^{c}	16.27^{c}	6.55^{b}	16.46^{a}	26.71^{a}	10.04^{cd}	19.79^{c}	4.70^{a}	3.85^{a}	4.18^{b}	11.79^{a}	11.91^{cd}	16.16^{bc}
		7.5 CS	2.79^{c}	3.34^{c}	1.81^{c}	18.19^{ab}	7.95^{a}	16.91^{a}	21.85^{c}	11.19^{bc}	21.62^{b}	4.16^{ab}	3.76^{a}	4.15^{b}	9.36^{b}	11.69^{cd}	13.48^{e}
		10 CS	3.44^{b}	7.91^{a}	3.14^{a}	18.29^{ab}	4.42^{d}	17.56^{a}	21.48^{c}	9.44^{d}	22.04^{ab}	4.04^{b}	1.65^{b}	4.52^{b}	8.41^{c}	12.72^{bc}	15.89^{c}
	120 DAT	CON	1.18^{c}	3.57^{d}	1.32^{c}	10.11^{b}	9.37^{c}	16.89^{d}	17.50^{d}	9.42^{b}	13.33^{c}	2.97^{b}	3.47^{a}	3.62^{c}	6.17^{d}	8.70^{d}	11.58^{d}
		NaCl	9.93^{a}	13.21^{a}	3.57^{a}	10.08^{b}	7.32^{e}	18.47^{c}	24.49^{c}	9.40^{b}	14.46^{a}	2.80^{b}	2.58^{bc}	4.01^{b}	7.76^{c}	13.26^{bc}	13.30^{b}
		1 MS	5.82^{b}	8.99^{c}	2.92^{b}	10.58^{b}	6.31^{f}	22.97^{b}	26.41^{b}	10.36^{ab}	13.56^{bc}	2.74^{b}	2.25^{c}	4.53^{a}	7.93^{bc}	14.10^{b}	11.98^{cd}
		2 MS	5.38^{b}	10.36^{bc}	2.89^{b}	10.29^{b}	8.43^{d}	19.59^{c}	30.72^{a}	10.54^{ab}	14.08^{ab}	3.01^{b}	2.97^{b}	3.93^{bc}	8.87^{a}	16.02^{a}	12.56^{bcd}
		7.5 CS	5.33^{b}	12.81^{a}	3.09^{b}	11.44^{b}	11.48^{b}	22.49^{b}	27.58^{b}	11.32^{a}	14.53^{a}	2.96^{b}	2.91^{b}	4.11^{b}	8.43^{ab}	15.47^{a}	14.35^{a}
		10 CS	5.52^{b}	10.82^{b}	2.89^{b}	13.96^{a}	12.45^{a}	25.44^{a}	26.27^{b}	9.78^{b}	11.86^{d}	3.59^{a}	3.39^{a}	4.14^{b}	8.42^{ab}	12.67^{c}	12.92^{bcd}
F. arundinacea	60 DAT	CON	1.26^{e}	0.32^{c}	1.38^{d}	3.06^{bcd}	2.35^{b}	4.15^{bc}	18.18^{a}	1.54^{bc}	15.87^{a}	2.71^{a}	1.40^{ab}	2.53^{b}	1.86^{a}	0.29^{a}	1.07^{b}
		NaCl	6.37^{bc}	1.23^{a}	1.79^{d}	3.65^{a}	2.21^{b}	2.06^{d}	12.26^{c}	2.01^{a}	5.56^{c}	2.58^{ab}	1.66^{a}	0.94^{c}	1.37^{b}	0.37^{a}	0.48^{c}
		1 MS	7.17^{a}	1.36^{a}	9.58^{b}	2.69^{d}	2.02^{b}	4.60^{ab}	14.05^{b}	1.74^{b}	12.64^{b}	2.44^{ab}	1.54^{a}	3.25^{a}	1.11^{bc}	0.32^{a}	1.03^{b}
		2 MS	3.95^{d}	0.47^{bc}	7.97^{c}	2.80^{cd}	3.05^{a}	3.63^{c}	10.76^{d}	1.66^{b}	13.24^{b}	2.57^{ab}	1.32^{cabc}	2.99^{ab}	0.98^{c}	0.34^{a}	0.95^{b}
		7.5 CS	5.88^{c}	1.00^{ab}	7.48^{c}	3.56^{ab}	2.61^{ab}	5.13^{a}	12.26^{c}	1.31^{cd}	12.56^{b}	2.59^{ab}	1.14^{bc}	2.68^{b}	1.17^{bc}	0.21^{a}	0.90^{b}
		10 CS	6.72^{ab}	1.00^{ab}	11.74^{a}	3.36^{abc}	2.61^{ab}	4.24^{bc}	12.36^{c}	1.18^{d}	16.51^{a}	1.91^{b}	1.02^{c}	2.51^{b}	1.46^{b}	0.20^{a}	1.51^{a}
	120 DAT	CON	0.92^{d}	0.22^{c}	1.28^{d}	2.72^{d}	1.43^{a}	4.29^{ab}	11.67^{ab}	1.79^{bc}	19.95^{a}	2.07^{c}	1.77^{bc}	3.32^{b}	1.12^{b}	0.37^{a}	1.64^{a}
		NaCl	8.01^{b}	0.52^{bc}	4.92^{c}	3.17^{c}	1.37^{a}	2.54^{d}	10.51^{bc}	2.26^{a}	9.74^{e}	2.50^{b}	1.99^{ab}	1.90^{d}	0.99^{bc}	0.38^{a}	0.75^{c}
		1 MS	1.11^{d}	0.26^{c}	1.44^{d}	3.14^{c}	1.89^{a}	4.25^{ab}	9.18^{c}	2.15^{ab}	15.44^{d}	2.99^{a}	2.26^{a}	3.78^{a}	0.75^{bc}	0.35^{a}	1.04^{bc}
		2 MS	6.97^{c}	0.99^{a}	7.30^{b}	2.61^{d}	1.50^{a}	3.12^{c}	10.55^{bc}	1.91^{ab}	16.77^{c}	2.48^{b}	1.37^{cd}	2.78^{c}	1.00^{b}	0.28^{a}	1.16^{b}
		7.5 CS	6.55^{c}	0.62^{b}	7.61^{b}	3.58^{b}	1.59^{a}	4.40^{a}	9.26^{c}	1.38^{c}	17.84^{b}	1.98^{c}	1.33^{cd}	2.26^{d}	0.64^{c}	0.21^{a}	0.99^{bc}
		10 CS	8.86^{a}	0.77^{ab}	8.11^{a}	4.50^{a}	1.30^{a}	3.99^{b}	12.14^{a}	1.47^{c}	19.66^{a}	1.68^{c}	1.27^{d}	2.08^{d}	1.55^{a}	0.25^{a}	1.73^{a}
